# **Evaluating the potential of semi-continuous itaconic acid fermentation by* Aspergillus terreus***: **operational profile and experiences**

**DOI:** 10.1007/s11274-023-03797-9

**Published:** 2023-10-16

**Authors:** Éva Hülber-Beyer, Katalin Bélafi-Bakó, Tamás Rózsenberszki, Péter Komáromy, Nándor Nemestóthy

**Affiliations:** Research Group on Bioengineering, Membrane Technology and Energetics, Faculty of Engineering, University of Pannonia, Egyetem str. 10, Veszprém, 8200 Hungary

**Keywords:** Accessory conidium, Aspergillus terreus, Itaconic acid, Semi-continuous fermentation

## Abstract

Itaconic acid is an important bio-based chemical. The present study aims to evaluate the applicability of semi-continuous fermentation technique for itaconic acid production by *Aspergillus terreus.* The fermentation is planned to be connected with bipolar membrane electrodialysis unit for acid recovery. This process allows the reuse of residual glucose from the effluent. Our particular attention was focused on the effect of glucose concentration. Two different glucose supplementation strategies were tested: constant glucose concentration in the refilling medium and adjusted glucose concentration in order to maintain a continuously high – 120 g/L – glucose concentration in the fermentor. The itaconic acid titre, yield and productivity for the 24 h time periods between draining/refilling interventions were investigated. The constantly high glucose concentration in the fermentor resulted in doubled biomass formation. The average itaconic acid titre was 32.9 ± 2.7 g/L. The producing strain formed numerous spores during semi-continuous fermentation that germinated continuously. Yield and volumetric productivity showed a periodic pattern during the procedure.

## Introduction

Itaconic acid (IA) is a promising bio-based chemical for the polymeric industry. It can be readily polymerized, co-polymerized with other monomers (Sollka and Lienkamp 2021), or converted to other precursors (Teleky and Vodnar 2019) presently produced from fossil feedstock. The most effective IA-producing organisms known today are some subspecies of *Aspergillus terreus* (Bafana and Pandey 2018). This filamentous fungus has a complex life cycle (Upshall et al. 1977; Lee et al. 2016). Its key metabolic pathways that lead to IA secretion have been explored and confirmed by genetic studies, and also targeted by genetic engineering (Boruta and Bizukojc 2017; Huang et al. 2014; Yu et al. 2023).

Bio-based platform chemicals are needed in larger quantities than e.g. drug precursors. Batch production thus requires reactors with large volume. Scaling up fermentation processes is difficult due to the vaguely predictable effects of changed environmental conditions i.e. hydraulic pressure, aeration efficiency, reduced mixing quality etc. (Schmidt 2005). In contrast, semi-continuous and continuous processes can be operated in a reduced-sized bioreactor with good productivity (Tikhomirova et al. 2018).

Bipolar membrane electrodialysis is a promising product recovery technique for IA. With this technique, the undissociated acid can be recovered along with a pure alkali solution and a potentially reusable diluate stream, in which non-polar compounds, such as sugars, are retained (Hülber-Beyer et al. 2021).

Our previous investigations on IA separation with this technique using binary and terciary model solutions have shown that the highest recovery ratio could be obtained with 33 g/L IA concentration, 5–33 g/L being the investigated range (Komáromy et al. 2020b; Rózsenberszki et al. 2020).

A promising way to produce IA may be the integration of a continuous fermentation procedure and product recovery with bipolar membrane electrodialysis. By nutrient recycling from the diluate, the integrated system may show good raw material utilisation and minimum water use.

A few research were conducted in the past to investigate continuous IA production with immobilized cells (Horitsu et al. 1983; Kautola et al. 1985; Welter 2000; Federici 1993) or with free mycelia (Kobayashi and Nakamura 1966; Rychtera and Wase 1981; Ju and Wang 1986; Kautola et al. 1990; Welter 2000). The achievable product concentrations in continuous processes were far behind the ones achieved in batch production (Krull et al. 2017), 0.15–20 g/L and 70–160 g/L respectively. Kobayashi and Nakamura (1966) described product inhibition during continuous IA fermentation with *A. terreus* K 26. They found that above 20 g/L IA the productivity linearly decreases with further IA concentration increase. This inhibition was less significant during batch cultivation and appeared only around 50 g/L.

After the last research on continuous technique with *A. terreus*, several new results were obtained in batch processes (Kuenz and Krull 2018) that encourage the re-evaluation of the previous findings. One of these is the positive effect of high sugar concentration − 12–20% (w/V) – on IA yield and accordingly on the achievable product concentration (Karaffa et al. 2015).

In a semi-continuous fermentation (SCF) a given portion of the broth is removed from the bioreactor regularly and replaced by fresh medium. The modifications are made in discrete steps. The process is therefore simple to implement. An increase in the frequency of volume exchanges and a decrease in the volume of broth changed on these occasions, would finally result in a real continuous process.

Semi-continuous and continuous fermentation processes are well established in case of single-cell organisms like yeasts and bacteria (Egli 1991; Ertugay and Hamamci 1997; Wanner and Egli 1990). The complex growth pattern and behaviour of *A. terreus* may cause difficulties to establish a (semi)continuous fermentation (Welter 2000). Nevertheless, our aim was to study this complicated process, to establish a semi-continuous procedure with high IA titer in the effluent, thus laying the foundation of the integrated fermentation and electrodialytic IA separation system.

## Materials and methods

### Microorganism and culture media

In this study, *A. terreus* NRRL 1960 served as the IA-producing strain. The microorganism was maintained on supplemented potato-glucose agar (Fluka™, Honeywell, USA) slants, into which 20 g/L sodium chloride and 10 g/L glucose monohydrate were added before sterilization (Komáromy et al. 2019).

For inoculation of the fermentor, freshly prepared conidium suspension was used. A surface culture was incubated for 7 days at 37 °C in a Roux culture flask with 185 cm^2^ agar surface. Conidia were harvested by washing the agar surface with 100 mL saline containing 0.1% (w/w) Tween-80. Spore concentration was determined with a Buerker counting chamber under a Zeiss microscope (PrimoStar, Zeiss AG, Germany). An amount of inoculum was added into the fermentor to reach 5·10^6^ spore/mL.

The fermentation medium was adopted from the study of Karaffa and co-workers (2015) and contained 150 g/L glucose. The pH was set to 3.0 before sterilization with 10% (w/w) sulphuric acid solution. The pH adjustment prevented browning of glucose during sterilization. The glass reactor vessel containing the complete medium was sterilized in an autoclave at 121 °C for 45 min.

The composition of the refilling medium for the two independent SCF experiments is shown in Table 1. It contained no added trace elements. Ammonium nitrate was added to prevent nitrogen limitation. The amount of magnesium and calcium was identical to the original fermentation medium, to maintain a constant amount in the fermentor. The biomass renewal is of great importance since there was no cell retention and calcium is an important element in fungal development and acid production (Pera and Callieri 1999).

**Table 1:** Refilling medium composition for semi-continuous fermentation experiments

*Component name*	SCF I.	SCF II.
Glucose	100 g/L	adjusted to reach 120 g/L (Karaffa et al. 2015)
KH_2_PO_4_	0.1 g/L	
NH_4_NO_3_	2.25 g/L	
MgSO_4_ × 7 H_2_O	1 g/L	
CaCl_2_ × 2 H_2_O	5 g/L	

The materials applied in this study were of analytical reagent grade, purchased from Merck, except for glucose which was the product of Hungrana Starch and Isoglucose Manufacturing and Trading Ltd.

### Equipment and procedures

The two experiments were carried out in a BIOSTAT® Bplus (Sartorius, Germany) bioreactor system. The reactor vessel had 1.5 L working volume. Two 6-blade Rushton turbines were applied on the central mixing shaft.

A series of batch experiments preceded the implementation of SCF in our laboratory (Nemestóthy et al. 2019, 2020; Komáromy et al. 2019) aiming to evaluate parameter – such as pH, DO, stirring rate – sensitivity of the producing strain. Preliminary experiments have also been conducted to work out practical steps involved in SCF.

Inoculum was added into the fermentor from a sterile flask. The spore suspension was filled in the flask in a laminar box. When the temperature, pH and dissolved oxygen level reached the required values (37 °C, 3.1 pH, 100% respectively), the spore suspension was injected into the fermentation medium.

The temperature was kept at 37 °C. A constant 1.33 VVM air inlet provided the oxygen supply for growth and production. Based on preliminary oxygen transfer rate measurements (data not shown), the stirring rate was set to 500 rpm. According to a previous study performed in our laboratory, this particular fungal strain tolerated this mixing speed and performed best under similar conditions in batch fermentation (Nemestóthy et al. 2019).

The applied pH control strategy was the one suggested by Hevekerl and co-workers (2014), who found that letting the pH sink below 2, then starting the control to keep 3.1 is optimal to achieve high final IA titre. 15% (w/w) NaOH solution was used for pH control.

Antifoam L-30 emulsion (Sigma) has been added automatically if needed.

The semi-continuous operation was started in the decelerating growth phase. Most of the acid is formed during this period (Rychtera and Wase 1981). The IA productivity is maximal in the 4–5. day of fermentation, according to our previous findings for batch cultivation (Komáromy et al. 2019). Drain/feed cycles were performed at 24-hour intervals. Fermentations were stopped after the alkali was not consumed for twelve hours.

In the 12 days long SCF I. experiment the semi-continuous operation started on the 5. day of the batch run-up phase. A constant glucose concentration of 100 g/L was applied in the refilling medium. Seven drain/feed cycles were performed, in which the exchanged volume was 100 mL (6.7%) on the first five occasions and 200 mL (13.4%) on the last two. The respective apparent dilution rates are 0.0035 h^− 1^ and 0.007 h^− 1^.

SCF II. lasted 10 days, with semi-continuous operation starting on day 4. During the semi-continuously operating part of the fermentation, the amount of glucose was changed dynamically in the refilling medium day by day as a function of consumption. In each cycle, the glucose concentration was readjusted to 120 g/L. This level was found to be the optimal initial glucose concentration in batch fermentations by Karaffa and co-workers (2015). During SCF II. six drain/feed cycles were completed, each taking up 200 mL (13.4%) volume change that corresponded to 0.007 h^− 1^ apparent dilution rate. Before interventions, 2 mL samples were taken and glucose levels were determined. From this we calculated the amount of sugar needed. The refilling medium was prepared from concentrated salt solutions (1–1 mL for 100 mL medium), 400 g/L glucose syrup and deionized water – each autoclaved previously and kept as stock solutions. The draining was performed when the refill medium was ready for use. After refilling, the actual sugar concentration in the reactor was determined in the fermentor from another sample.

### Analytical methods

IA, glucose, and dry biomass concentrations were evaluated from the drained broth. To check for microbial contamination, a small amount of sterile sample was taken directly from the fermentor.

IA production was monitored by HPLC on a YL9100-type device (Young Lin Instrument Co., Ltd., South Korea) containing a Hamilton PRP×300 HPLC column (150 × 4.1 mm, 7 μm) as well as a UV/VIS detector, at 210 nm detection wavelength. The analytical method employed a gradient elution, where the moving phase was comprised of A (1 mM H_2_SO_4_) and B (methanol) solutions (0 min–100% A; 1 min – 70% A, 30% B; 6 min – 70% A, 30% B; 8 min – 100% A). The samples were filtered on a 0.2 μm pore-sized Nylon syringe filter and thereafter diluted a 1000-fold using 1 mM sulphuric acid. The injection volume was 100 µL.

Glucose concentration was determined by the dinitrosalicylic acid method, after appropriate sample dilution (Jain et al. 2020).

To obtain data on dry biomass concentration (BDW), 3 × 15 mL of well-mixed broth was centrifuged at 10 000 rpm for 15 min (20 °C). After removing the supernatant, equal amount of sterile water was added, shaken thoroughly to resuspend the settled biomass and thus remove the residual solutes between the mycelia, and centrifuged again. The washing procedure was carried out twice and then the biomass was dried at 80 °C until constant weight. IA, glucose and BDW measurements were performed in triplicates.

For microscopic analysis, a Zeiss PrimoStar microscope was used with a phase contrast equipment, at one thousand-fold magnification.

The spread plate method was used to check for microbial contamination. The samples were spread on PDA plates and incubated for one week at 37 °C. The plates were examined on a daily basis for colonies with morphology different from *A. terreus*.

## Results

The presented SCF fermentations varied in the glucose content of the refilling medium. In SCF I., a constant glucose concentration of 100 g/L was applied while in SCF II., the glucose concentration was changed dynamically day by day to readjust the sugar content in the fermentation broth to 120 g/L.

The experiments were followed by the various analytical methods for monitoring IA, glucose and BDW concentrations. Detailed evaluation of the experiments and the important qualitative observations are discussed in two separate subsections.

In all cases morphology showed loose, fluffy mycelium form with no complex-shape structures. No microbial contamination was detected in any of the samples tested.

### Operational profile

Fermentations started with a run-up similar to batch procedures. Semi-continuous operation was started in the decelerating growth phase, during SCF (I) and SCF (II) on day 5 and day 4, respectively.

**Fig. 1:**
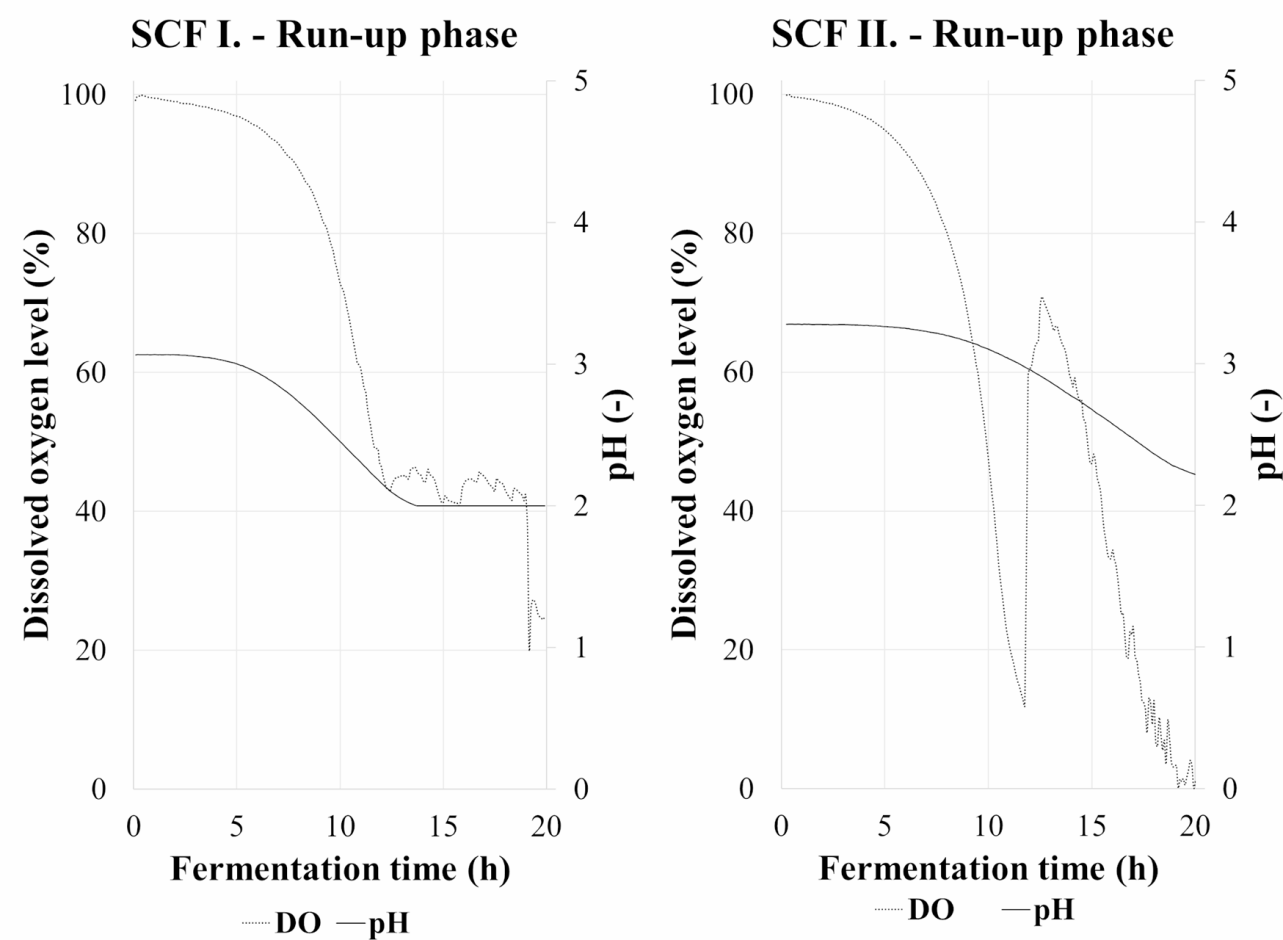
Changes in the level of dissolved oxygen and pH during the run-up phase of **(a)** SCF I and** (b)** SCF II. The black arrow indicates a serious foaming event when a significant portion of biomass was transferred in the foam

In the first 20 h of the fermentations, the dissolved oxygen level decreased rapidly along with the pH. During that time a minimal amount of samples were taken for glucose and IA determination. The increase in biomass dry weight could not be followed from such small volume. Nevertheless, the volumetric glucose consumption was low (Table 2) indicating low biomass concentration. The measured data of the run-up phase are presented in Fig. 1.

**Table 2:** Acceleration of instantaneous volumetric glucose consumption during the run-up phase in SCF II

SCF II.		
*Fermentation time*	*Glucose concentration*	*Rate of glucose consumption*
0 h	149.3 g/L	
6 h	148.6 g/L	0.12 g/L/h
19 h	146.7 g/L	0.15 g/L/h
24.5 h	144.4 g/L	0.42 g/L/h
35.5 h	138.1 g/L	0.57 g/L/h
48.5 h	129.2 g/L	0.68 g/L/h
71 h	108.4 g/L	0.92 g/L/h

In that early stage, no IA was detectable in the broth so the acid did not contribute to the development of low pH. However, the automatic alkali addition correlated with acid production during IA production.

**Fig. 2:**
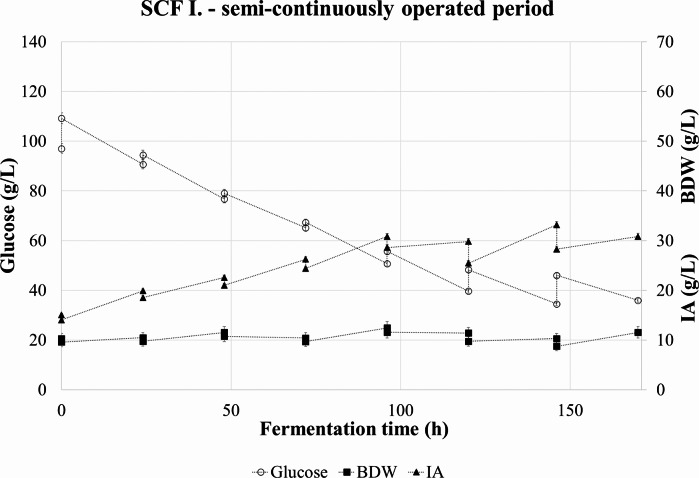
Concentration of glucose, itaconic acid, and biomass dry weight during semi-continuous operation in SCF I

The measured concentration data of the SCF I. at the beginning and during semi-continuous operation is shown in Fig. 2.

The glucose concentration showed a decreasing tendency, while the IA concentration slowly increased. The maximal product concentration – 33.2 g/L IA – was measured in the sixth cycle. During the semi-continuous production the average IA concentration was 27.6 ± 4.9 g/L. The BDW profile had an unusual shape, most probably caused by the foaming and mycelial adherence to headspace surfaces. The average in the process was 11.2 ± 0,8 g/L BDW, with no evident tendency of growth or decline. In the last two cycles, the exchanged volume was doubled. This is noticeable in the rate of increase in the glucose concentration and decrease in the IA concentration after refilling. The fungus reproduced its mass even at higher dilution rate. The higher dilution step resulted in a 2–3 h-long break in alkali addition that did not occur at lower dilution. The slope of alkali addition was rapidly restored to its original value when dosing has started again.

**Fig. 3:**
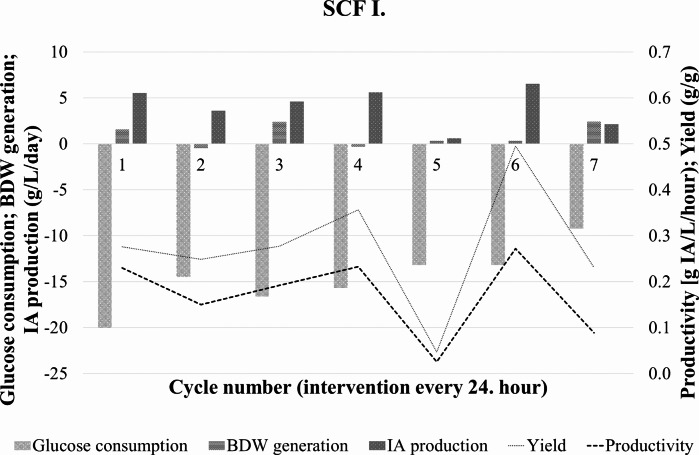
Change in glucose, itaconic acid, and biomass dry weight concentrations during the semi-continuous operation in SCF I. Itaconic acid yield and average productivity for the 24 h cycles are indicated with different dashed lines

The consumed glucose, produced IA and biomass dry weight in each cycle are presented in Fig. 3, together with the calculated volumetic productivity and product yield data for the 24 h periods between interventions.

The productivity had three peaks during the first, the fourth, and the sixth cycle – 0.23, 0.23, and 0.27 g ITA/L/h, respectively. IA yield showed similarly three maxima in these cycles, namely 0.28, 0.35, and 0.50 g ITA/g glucose. An apparent periodicity could be observed during the process.

The volumetric glucose consumption rate during the production phase averaged around 0.68 g/L/h with a minimum of 0.55 g/L/h and a maximum of 0.83 g/L/h. (Table 3.) The average specific glucose consumption was 0.07 g glucose/L/h/g BDW.

**Table 3:** Volumetric glucose consumption rate during the production phase in SCF I

SCF I.	
*Period*	*Rate of glucose consumption*
1. − 2.	0.83 g/L/h
2. − 3.	0.78 g/L/h
3. − 4.	0.60 g/L/h
4. − 5.	0.69 g/L/h
5. − 6.	0.65 g/L/h
6. − 7.	0.55 g/L/h

**Fig. 4:**
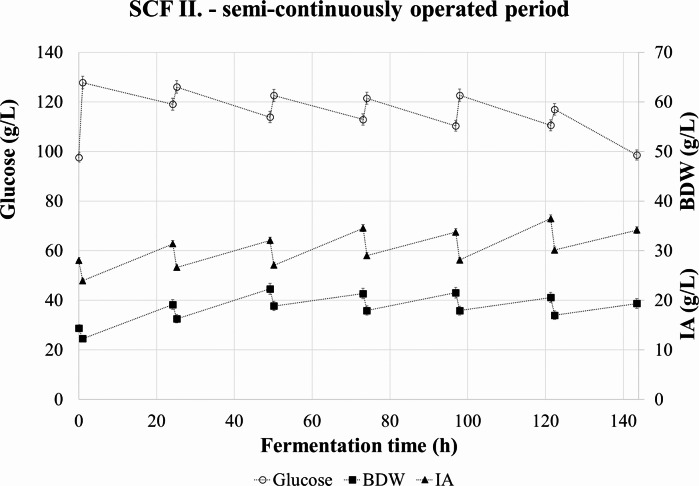
Concentration of glucose, itaconic acid, and biomass dry weight during the semi-continuous operation in SCF II

In the second experiment, glucose concentration was reset to a higher level in every cycle. The measured concentration data of the SCF II. at the starting of and during semi-continuous operation is shown in Fig. 4.

The average IA titre in drained broth volumes was 32.9 ± 2.7 g/L, with a maximum of 36.5 in the fifth cycle. The average BDW was 19.8 ± 2.7 g/L with a slow-growing tendency. The suspended BDW data gave a rather balanced shape. However, the doubling of the cell mass resulted in a thick broth from which it was hard to filter off or centrifuge the mycelia.

**Fig. 5:**
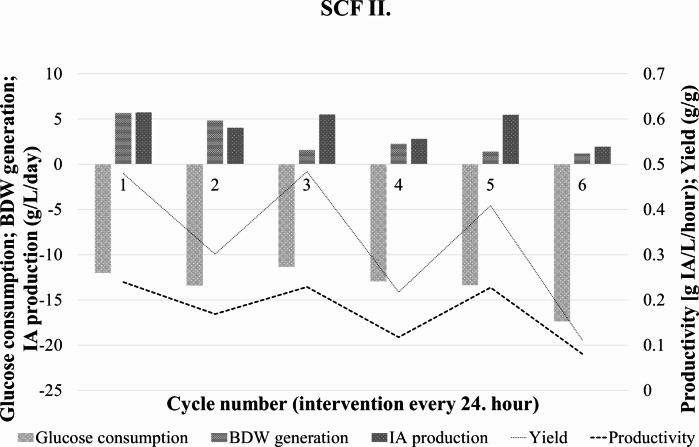
Change in glucose, itaconic acid, and biomass dry weight concentrations during the semi-continuous operation in SCF II. Itaconic acid yield and average productivity for the 24 h cycles are indicated with different dashed lines

The consumed glucose, produced IA and BDW in each cycle are presented in Fig. 5. Dashed lines indicate the calculated volumetric productivity and IA yield for every 24 h periods during SCF II.

The apparent periodicity was even more pronounced in this case than in the first experiment. Productivity had three peaks in the first, third, and fifth cycles of 0.24, 0.23, and 0.23 g ITA/L/h, respectively, and the lowest value was 0.08 g ITA/L/h. The yield followed a similar pattern with 0.43, 0.43, and 0.41 g ITA/ g glucose, respectively.

The volumetric glucose consumption rate during the production phase in SCF II. averaged around 0.56 g/L/h with a minimum of 0.47 g/L/h and a maximum of 0.72 g/L/h. (Table 4.) The average specific glucose consumption was 0.04 g glucose/L/h/g BDW. This is 37% less than in SCF I.

**Table 4:** Volumetric glucose consumption rate during the production phase in SCF II

SCF II.	
*Period*	*Rate of glucose consumption*
1. − 2.	0.56 g/L/h
2. − 3.	0.47 g/L/h
3. − 4.	0.54 g/L/h
4. − 5.	0.56 g/L/h
5. − 6.	0.72 g/L/h

### Special growth of *Aspergillus terreus* NRRL 1960 during SCFs

During the run-up period of the fermentation, the broth was prone to foaming. The foam consisted mainly of biomass, therefore, when it occurred, a sharp increase in dissolved oxygen level was observed (black arrow on Fig. 1b). The addition of an anti-foaming agent proved to be moderately efficient, since more than tenfold of the recommended dosage was needed in order to dampen foaming.

**Fig. 6:**
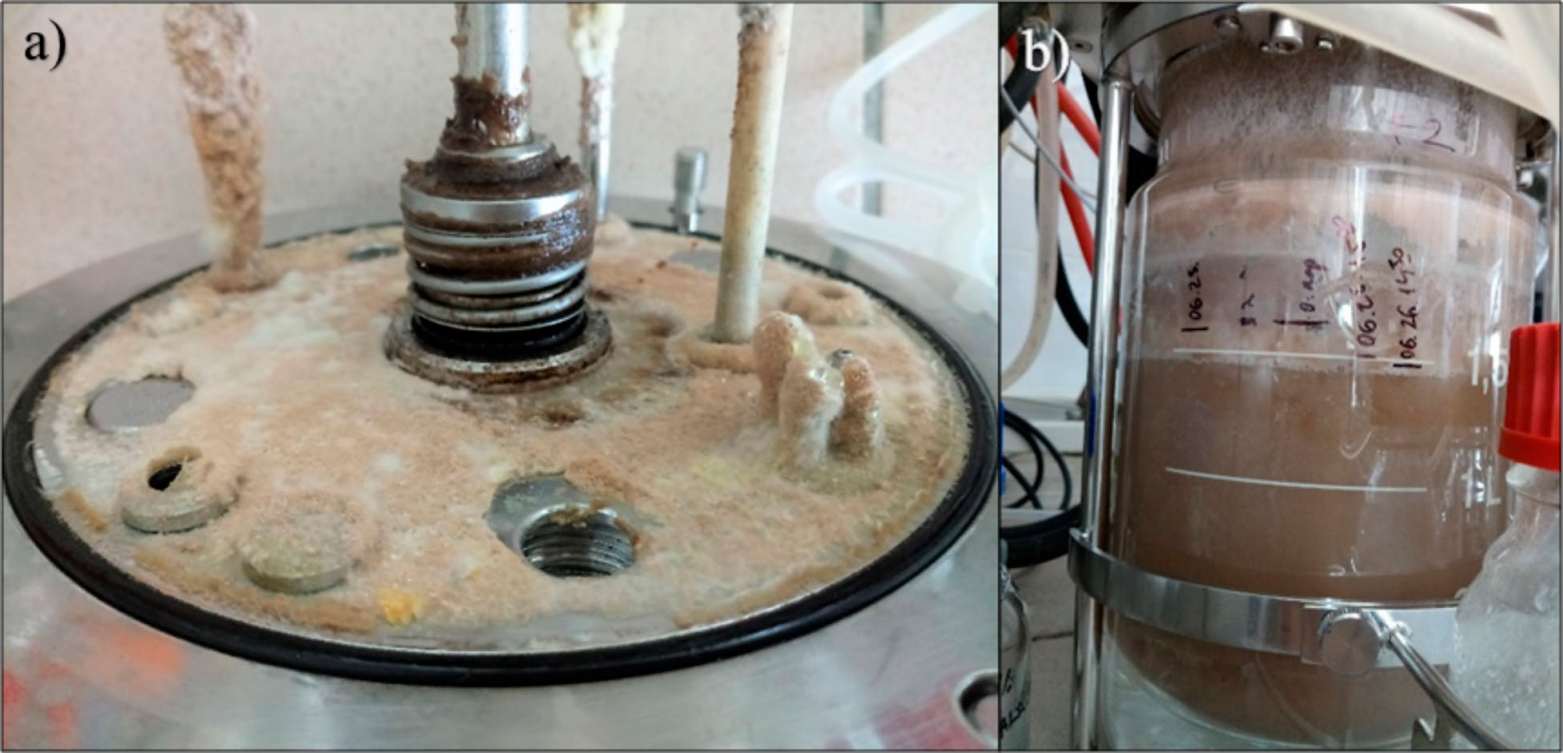
Fungal biomass coating on the lid of the bioreactor **(a)** and the glass reactor vessel **(b)**. The cinnamon-colored area shows heavy sporulation i.e. conidia formation. (Pictures were taken after disassembly.)

On every surface of the headspace in the bioreactor, a biomass layer built up and thickened during the process that was fed continuously by the aerosol formed because of intensive aeration (Fig. 6). In case of SCF II., the biomass layer on the headspace surfaces grew even more intensively compared to SCF I.

The biomass adhering to the headspace surfaces did not contribute to IA production but used the nutrients – e.g. glucose – from the broth. Thus, important indicators such as the mass-balance and biomass yield could not be determined precisely.

However, the suspended biomass dry weight (BDW) could be measured from the drained broth samples and the data was used for calculations as that portion of biomass is responsible for the IA production.

**Fig. 7:**
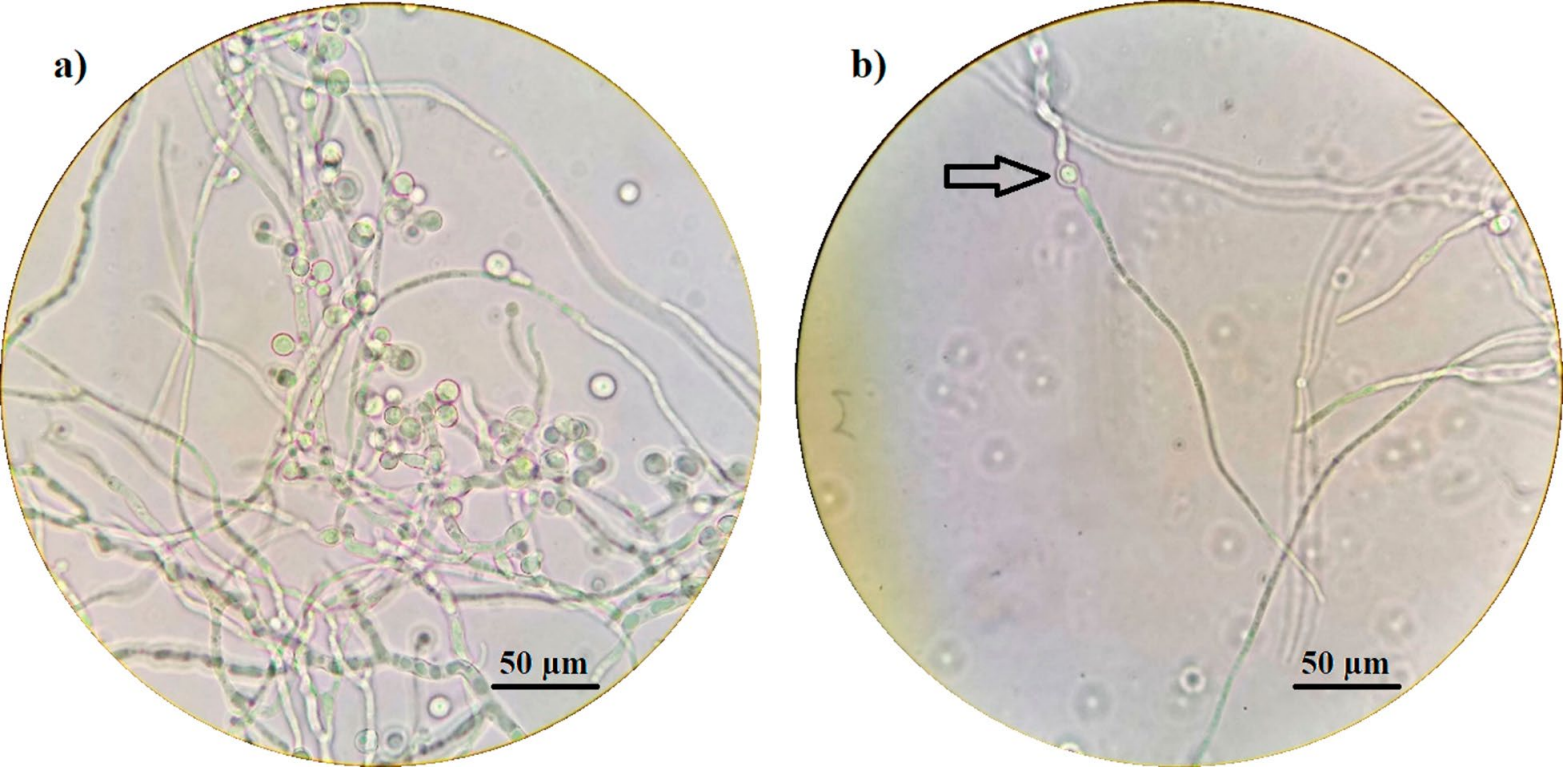
Sample from the SCF II. fifth draining: **(a)** accessory conidia on the fungal mycelium; **(b)** germinating accessory conidium in the same sample (1000x magnification)

Phase-contrast microscopic investigations during both fermentations showed intense aleuriospore-formation during the semi-continuously operating phase. These spores tend to germinate thus not only the hyphal vegetative growth occurs, but also an additional spore originated growth (Fig. 7).

## Discussion

In this study, two successful SCFs were presented and evaluated. The results showed the feasibility of the procedure. We achieved a higher average product concentration in the cell-free effluent than those achieved by Richtera and Wase (1981) – 7.8 g/L or Welter (2000) – 0.15–0.3 g/L in both implementation – 27.6 ± 4.9 g/l and 32.9 ± 2.7 g/l in SCF I. and SCF II., respectively. The cell free effluent was collected and can be subjected to bipolar membrane electrodialysis for IA recovery. The findings of the process on real fermentation broth are essential for development of an integrated continuous IA fermentation and electrodialytic recovery process. The applied apparent dilution rate – 0.007 h^− 1^ – was an order of magnitude lower than in any study on continuous IA production.

Karaffa et al. (2015) found, that manganese ions have an inhibiting effect on IA production with *A. terreus*. However, in our experiments, manganese ion concentration was not controlled. According to their work, the highest achievable IA titre would have been less than 50 g/L if the fermentations had been allowed to reach their ends as batch processes. The product concentrations in the drained broth samples are promising when compared to this. However, with the control of manganese ion concentration more than 90 g/l would be achievable. Our glucose substrate was of food grade and its manganese content was not tested. The achievable IA titre may be improved with strict manganese control.

The apparent periodicity of the process showing maxima and minima for both the volumetric productivity and product yield during the 24-hour long intervals are puzzling. A possible explanation can be the complex growth of *A. terreus*. We observed that during the semi-continuous operation numerous submerged spores are formed that tend to germinate. Thus, two growth paths are present during the process. Changes in dominant growth path might have a correlation with the periodicity of productivity and yield. But as a side effect, the production of accessory conidia raises the possibility that the mycelium in the fermentor may be continuously renewed, thus the ageing of the culture can be prevented, or at least delayed.

The maximum measured volumetric productivity of 0.27 g/L/h was less than those reported previously by Kobayashi and Nakamura (1966) or Rychtera and Wase (1981), 0.48 g/L/h, and 0.32 g/L/h, respectively.

The theoretical maximum IA yield calculated from the metabolic pathway is 0.72 g ITA/g glucose – in that case, all of the consumed glucose goes through the IA pathway. In these SCF processes, no cell retention was applied, so biomass reproduction was an important issue for long-term operation, i.e. the product yield could only approach the theoretical maximum, but not reach it. Thus, the 0.50 g ITA/g glucose product yield obtained in SCF I. is promising.

The constantly high glucose concentration in SCF-II resulted in almost doubled biomass production, higher product titre and better yield than those obtained in SCF-I, but productivity was not improved considerably. The high biomass content hinders the clarification of the broth (i.e. separation of biomass by centrifugation or filtration), thus in future experiments, the correlation of sugar concentration and phosphate supplementation shall be evaluated. Further SCF experiments should be carried out to find the optimum ratio of phosphate and sugar in the refilling medium to reach high productivity, high yield, and minimum biomass concentration. Our findings also indicates that cell retention might not be the way of an effective process. The amount of phosphate source was the same in both methods, this shows that glucose in excess had built in to the biomass more effectively.

The average specific volumetric glucose consumption rate during SCF II. lowered by 37% compared to the SCF I., which was performed with glucose levels below 100 g/L. This suggest substrate inhibition during continuous operation. The finding that increasing glucose concentration up to 120 g/L improves yield (Karaffa et al. 2015) may have an effect on the germination of conidia used in inoculum rather than on the fungal mycelial IA production itself.

It should be noted that macro-morphology of the *A. terreus* is considered as an important parameter during fermentation. Gao and co-workers (2014) found that a pelleted structure is favoured over the loose mycelia in order for achieving higher IA yield. In our case, the loose mycelium form was dominant. Changing the geometry of impellers to reduce shearing forces may allow the formation of pellets. However, Herrán and collaborators (2010) came to an interesting result when applying sonication for *A. terreus* fermentation – though their target product was lovastatin, another industrially important metabolite of the strain. They concluded that sonication disrupted the pellets and improved the rheology of the broth but had no significant effect on fungal growth and on yield.

Another issue resulting from the special growth of *A. terreus* is the adherence of the fungus to surfaces thus making difficult to calculate a precise mass-balance. Besides of improving oxygenation technique to avoid intense bubble formation (Komáromy et al. 2020a), the molecular mechanisms behind the phenomenon should be reviewed and clarified, and genetic engineering tools may be deployed.

## Conclusion

The semi-continuous cultivations of *A. terreus* resulted higher IA concentrations than those achieved in formerly published continuous fermentations.

In our previous investigations on IA recovery by bipolar membrane electrodialysis, we found that an IA concentration of 33 g/L can be recovered effectively. The achieved IA titres in SCF I. and SCF II., 27.6 ± 4.9 g/l and 32.9 ± 2.7 g/l respectively, are promising in the context of developing an integrated fermentation-electrodialysis system.

In the two experiments different glucose refilling strategies were applied. The readjustment of glucose concentration to 120 g/L in every cycle resulted in a doubled biomass content compared to the refilling with constant 100 g/L glucose level in the medium. The higher biomass, though, consumed glucose on 37% lower rate suggesting that high glucose level has an inhibitory effect on the process.

During the processes, extensive submerged spore formation was observed, that germinated simultaneously with hyphal growth. The presence of germinating spores may ensure the renewal of biomass and the aging of the culture may be delayed.

In future semi-continuous experiments, the manganese ion content shell be strictly controlled. Since macro-morphology of the fungus is an important parameter, an aeration and stirring technique should be applied that allows the formation of pellets rather than loose mycelium.

## Data Availability

The datasets generated during and/or analysed during the current study are available from the corresponding author on reasonable request.
